# Prophylactic Intramedullary Nailing Following Femoral Lengthening in Congenital Femoral Deficiency: A Retrospective Comparative Study

**DOI:** 10.3390/jcm15218564

**Published:** 2026-11-03

**Authors:** Jędrzej Tschurl, Tomasz Kotwicki, Piotr Janusz, Milud Shadi

**Affiliations:** 1 Poznan University of Medical Sciences, Doctoral School, 60-812 Poznan, Poland; 2 Poznan University of Medical Sciences, Department of Spine Disorders and Pediatric Orthopedics, 61-545 Poznan, Poland

**Keywords:** femoral lengthening, congenital femoral deficiency, regenerate fracture

## Abstract

**Background:** Fracture of newly formed bone (the regenerate) after EF (External Fixator) removal is a serious complication of bone lengthening in Congenital Femoral Deficiency (CFD). This study aimed to determine whether prophylactic intramedullary nailing (IMN) at the time of EF removal reduces the risk of femoral fractures in CFD and to evaluate its safety. **Methods:** This retrospective comparative study included 53 CFD patients who underwent femoral lengthening (44 in the IMN group, 9 in the historical no-IMN group). We analyzed the incidence of fractures, infections, and unplanned surgeries. The predictive value of Fischgrund principles and Li classification for fracture risk was evaluated. **Results:** The fracture rate was 11.3% (6/53). The IMN group had fewer fractures (6.8%) versus the no-IMN group (33.3%, *p* = 0.054). Surgical treatment risk for femoral fracture was significantly lower in the IMN group (2.3% vs. 33.3%, *p* < 0.05). Despite complications in the IMN group (4.5% infection rate, 4.5% nail malposition rate, 2.3% hip dislocation), the rate of unplanned surgery was lower than in the no-IMN group (11.4% vs. 33.3%, *p* = 0.12). Associations between regenerate morphology and subsequent fracture occurrence were observed only in the historical no-IMN group. Prophylactic nailing may provide mechanical support in patients with less mature regenerate. **Conclusions:** In this retrospective study with a historical comparison group (no-IMN), prophylactic intramedullary nailing was associated with fewer fractures requiring surgery after femoral lengthening with EF in CFD.

## 1. Introduction

Congenital Femoral Deficiency (CFD) is a rare (incidence rate of 1–2 cases per 100,000 births) congenital disorder frequently managed with multistage treatment comprising hip joint reconstruction surgery, correction of femoral deformity and femoral lengthening [1,2,3]. Standard method of bone lengthening is distraction osteogenesis using an external fixator (EF) [4]. Fractures of newly formed bone (the regenerate) after EF removal remain one of the most serious complications of bone lengthening procedures [5,6]. Such fractures may happen during short-term follow-up and cause limb-length loss or axial deviation that require an additional, technically demanding surgery, which could impact quality of life and treatment satisfaction [7,8].

Prophylactic intramedullary nailing performed after EF removal is one method proposed to protect against such complications [9,10,11,12]. We have incorporated intramedullary nailing after EF removal in CFD in our department’s treatment protocol. However, concerns remain regarding the risk of infection and complications from additional surgical procedures.

This study aimed to report outcomes and complications of prophylactic femoral intramedullary nailing done at the time of EF removal. We have compared patients treated with this method with historical data of patients treated without prophylactic intramedullary nailing. We report whether prophylactic nailing reduces the odds of femoral fracture and presented our protocol to prevent femoral fractures in CFD lengthening.

## 2. Materials and Methods

### 2.1. Material

The Institutional Review Board of Poznan University of Medical Sciences approved this study, as it was not an experimental research study (KB 832/20, on 4 November 2020).

Table 1 shows patient characteristics. This retrospective study identified 66 consecutive patients who underwent femoral lengthening with EF between 2015 and 2025 in a single department. The etiology of shortening was CFD in 53 cases, and other etiologies (post-traumatic, post-septic, achondroplasia) in 13 cases. After excluding patients with other etiologies (post-traumatic, post-septic, and achondroplasia) to ensure a homogeneous sample, a total of 53 patients with Congenital Femoral Deficiency who underwent single-segment femoral lengthening were included. All patients had no previous history of femoral lengthening. Before prophylactic IMN became our preferred treatment approach in 2017, nine consecutive patients with CFD underwent EF removal without subsequent intramedullary nailing (no-IMN group). Since then, 44 patients underwent prophylactic intramedullary nailing at the time of external fixator removal (IMN group). Thus, the no-IMN group represents a historical comparison cohort rather than a control group. The adoption of prophylactic IMN coincided with a shift toward monolateral external fixation, introducing potential confounding by treatment period and EF type. Patients with any type of EF (monolateral, ring/Ilizarov frame, or spatial frame) were included. In the final analysis, we included 43 patients treated with the Monolateral Rail System (MRS) and 10 with the Ilizarov/ring frame.

Patients were treated in a single department by a team experienced in limb lengthening. The initial distraction rate was 1 mm/day, subsequently adjusted based on ultrasound evaluation of the bone regenerate. The timing of EF removal was determined by the surgeon and based on regenerate quality evaluated on radiograms, clinical assessment of pin sites, and the psychosocial aspects of prolonged EF use in children. Prophylactic femoral intramedullary nailing was performed at the time of EF removal using a Rush nail or a single Titanium Elastic Nail (TEN). The choice between a Rush nail and a single TEN was based on femoral canal morphology and surgeon experience—whenever possible, a Rush nail of at least 4.0 mm diameter was used. In the IMN group, we used a Rush nail in 26 patients and a single TEN in 18 patients. Intramedullary implants were removed after one year.

### 2.2. Surgical Technique and Postoperative Protocol

EF removal is performed in the operating room under spinal anesthesia. After removal of the fixator, pins, and wires, the pin wounds are cleaned, debrided, and disinfected. When prophylactic intramedullary nailing is planned, the entire limb is scrubbed and draped. The thigh is covered with sterile iodine surgical film to protect pin sites. The femoral entry point at the tip of the greater trochanter is identified using a C-arm. A 2 cm skin incision is made over the greater trochanter. The nail’s diameter and length are verified using a C-arm. Whenever possible, given femoral shaft anatomy, a Rush nail of at least 4.0 mm diameter is preferred. In cases of a narrow canal or shaft deformation, a single TEN is used. The nail is bent to match the femoral shaft’s contour. The distal end of the nail is further bent to facilitate passage through the medullary canal. An awl is used to create the entry point at the tip of the greater trochanter. The nail is advanced antegrade from the greater trochanter to a level just above the distal femoral physis. No reaming is performed to facilitate nail passage. The nail is advanced using oscillating maneuvers or gentle tapping with an orthopedic mallet, under C-arm guidance. The wound is closed in a layered fashion. Pin wounds are packed with a gentamicin-collagen sponge. After surgery, patients can mobilize with full weight bearing, using crutches to assist with pain and balance. Crutches are discontinued at the 2-week follow-up visit.

### 2.3. Evaluated Parameters and Radiographic Assessment

Medical histories and radiographs of all patients were reviewed before and after femoral lengthening, at intramedullary nail removal, and at the last follow-up. Standing long-cassette radiograms were used for radiographic analysis. Femoral lengthening parameters (achieved lengthening, Ex-Fix Index—days with EF/achieved lengthening) and radiographic regenerate quality parameters were assessed at EF removal [13,14]. In all patients, radiographic assessment of bone regenerate quality was performed using radiographs obtained one day before EF removal. The shape of the regenerate was classified according to Ru Li and assigned to one of 5 shapes [13]. The number of cortices was assessed on radiographs prior to EF removal and classified as sufficient (>=3) or insufficient (<3) according to Fischgrund [14]. Patients who sustained a femoral fracture after fixator removal were identified. Fracture type was classified according to Kenwright and Simpson [7]. Complications during and after femoral lengthening, unplanned surgery, and femoral fracture treatment were assessed.

The main researcher (JT) gathered all data. Statistical analysis was performed using JAMOVI software (version 2.6.45.0). Significance was determined as *p* < 0.05. Grammarly (version 1.205.1) and Google Gemini AI were used for language editing.

## 3. Results

### 3.1. Femoral Fractures

#### 3.1.1. Fracture Prevention

Overall, there were 6 femoral fractures in our study (6/53, 11.3%). There were no statistically significant differences in sex and age between fractured and non-fractured patients (Mann-Whitney U, *p* > 0.05). In this homogeneous CFD cohort, we observed 3 fractures (6.8%) in the IMN group versus 3 fractures (33.3%) in the no-IMN group. Although the total fracture incidence showed a strong descriptive trend toward reduction in the IMN group (Odds Ratio OR = 6.83, 95% Confidence Intervals CI 1.11–42.0; ORs and their 95% confidence intervals were calculated using the sample odds ratio and Wald method), it did not reach statistical significance (Fisher’s exact test, *p* = 0.054).

#### 3.1.2. Reduction in Fractures Requiring Surgical Intervention

A statistically significant difference was observed in fracture severity and management. In the no-IMN group, all 3 fractures (100%) were displaced or associated with axial deformity, necessitating unplanned secondary surgical interventions. In contrast, in the IMN group, the nail provided structural protection; two fractures were non-traumatic screw-track stable fractures that healed successfully with conservative non-surgical treatment (6 weeks of non-weight bearing). Only 1 fracture in the IMN group required surgical revision. Consequently, the risk of requiring surgical treatment for a femoral fracture was significantly lower in patients with prophylactic intramedullary nailing (2.3% vs. 33.3%, Fisher’s exact test, *p* = 0.013, OR = 21.5, 95% CI 1.91–242).

Within the IMN group, Rush nails were used in 26 cases (10 females and 16 males, mean age 9.3 years) and a single TEN was utilized in 18 cases (9 females and 9 males, mean age 8.0 years). The mean nail diameter was significantly larger in the Rush nail group compared to the single TEN group (4.24 mm vs. 3.61 mm; U Mann-Whitney, *p* < 0.05). However, the mean percentage of canal fill at the narrowest point (46.9% vs. 51.0%, respectively) did not differ significantly between the groups (U Mann-Whitney, *p* = 0.427). No statistically significant differences in age or sex distribution were observed between the Rush nail and single TEN groups. Furthermore, no statistically significant difference in fracture occurrence based on nail type was noted: 1/18 (5.6%) in the single TEN group versus 2/26 (7.7%) in the Rush nail group (Fisher’s exact test, *p* = 1.0). Likewise, there was no statistically significant difference regarding fractures requiring surgical intervention: 1/18 (5.6%) in the single TEN group versus 0/26 (0%) in the Rush nail group (Fisher’s exact test, *p* = 0.409). Notably, however, the only fracture requiring surgical revision occurred in the single TEN group.

**Figure 1:**
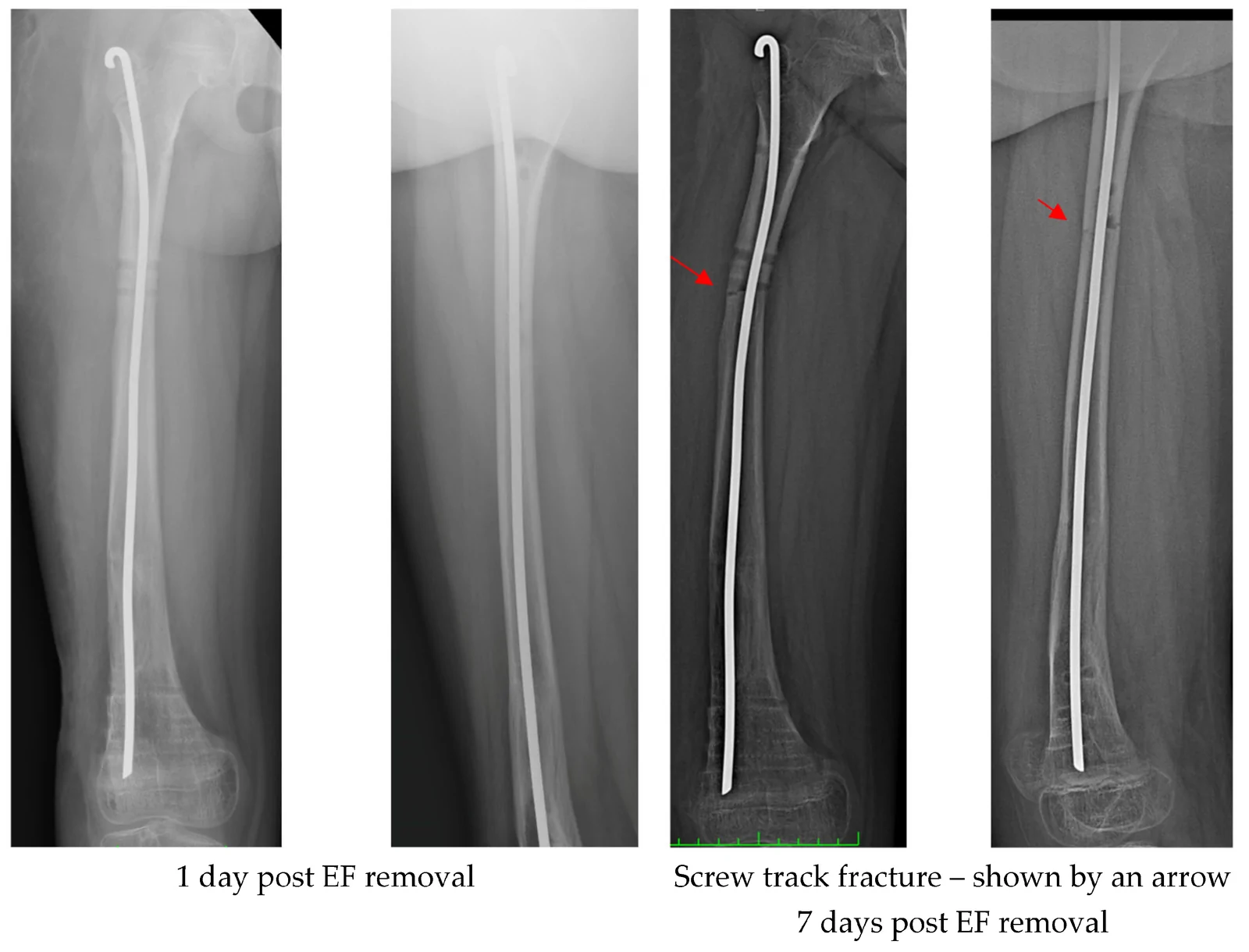
Example fracture in the IMN group: patient JJ. On the left femur 1 day after External Fixator (EF) removal with prophylactic intramedullary nailing. On the right screw track type fracture marked by an arrow, without loss of lengthening and angular deformity. Healed well with non-surgical treatment.

Out of 3 fractures in the IMN group, two fractures were due to low-energy injury, occurring during normal weight-bearing with crutches during the first 2 weeks after EF removal (example shown in Figure 1). Both fractures were classified as screw track type according to Kenwright and Simpson [7]. Neither patient required surgery; 6 weeks of non-weight bearing with crutches revealed sufficient to achieve bone healing. No loss of femur length (>5 mm) or angulation (>5 deg.) was observed in these two children.

The third fracture of the IMN group was due to a regular trauma during trampoline activity at 4 months post EF removal. This was a screw track type [7]. This fracture healed within 12 weeks following surgical treatment comprising closed reduction, replacement of the TEN nail with a Rush nail, and external fixation using a monolateral fixator. Table 2 summarizes the IMN group fracture cases.

**Figure 2:**
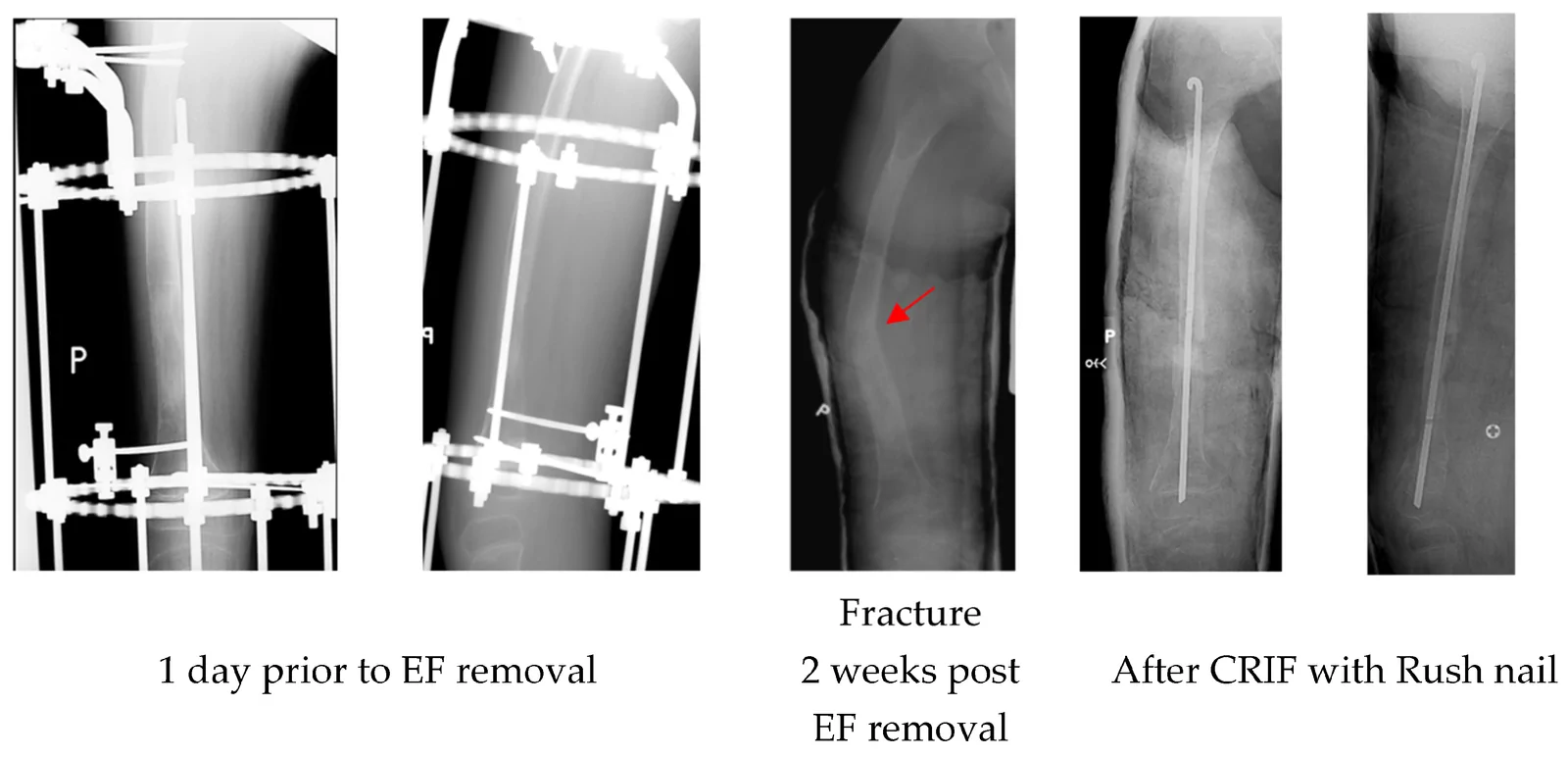
Example of fracture in the no-IMN group: patient ŁS. On the left femur 1 day prior to External Fixator removal. In the middle, junctional type fracture, marked by an arrow, 2 weeks after EF removal. On the right, after closed reduction with internal fixation (Rush nail) and casting.

All 3 fractures in the no-IMN group were due to low-energy injury and occurred during prescribed restricted weight-bearing activity, protected with crutches during the first 2 weeks after EF removal (example shown in Figure 2). Fractures were located within the regenerate or at the junctional level [7]. All required surgical treatment due to femur shortening or angulation (Table 3) using the intramedullary nailing: 2/3 with closed reduction and internal fixation (CRIF) using Rush nail and casting, and one initially with open reduction and internal fixation (ORIF) using plate (which failed), then with ORIF using femoral nail. Finally, all fractures healed without femur length loss or axial deviation, resulting in 4 unplanned surgeries in the no-IMN group. We have observed correct knee range of motion, without extension and flexion deficits in these patients.

### 3.2. Femoral Lengthening Parameters

Femoral lengthening parameters did not differ significantly between the IMN and no-IMN groups, or between fractured and non-fractured patients, as shown in Table 4 and Table 5. There appears to be a trend toward greater lengthening in patients with fractures; however, this was not statistically significant. Because EF type varied across groups, we performed additional statistical analyses to assess its impact on fracture occurrence and treatment. In the MRS subgroup, there were 5 fractures (5/43, 11.6%), and in the ring fixator subgroup, there was 1 fracture (1/10, 10%)—there was no statistically significant difference (Fisher’s exact test, *p* > 0.05). Similarly, there was no statistically significant difference in surgically treated fractures (6.9% vs. 10%, Fisher’s exact test, *p* > 0.05). Lengthening parameters did not differ significantly by EF type (*p* > 0.05). No statistically significant association was detected between EF type and fracture occurrence or the need for fracture-related surgery. However, the small and imbalanced subgroups limit the ability of this analysis to exclude a clinically relevant effect of EF type.

### 3.3. Knee Joint Range of Motion

Patients presented knee flexion deficit at EF removal, as shown in Table 6. No extension deficit was noted in both groups. There was no statistically significant difference, between the IMN versus the no-IMN group, as well as the fractured versus the non-fractured patients as comes to knee flexion and extension at the time of EF removal. Knee flexion improved significantly between EF removal and nail removal (36 degrees vs. 109 degrees, *p* < 0.001).

### 3.4. Bone Regenerate Quality

There was a substantial rate of patients with poor regenerate morphology (<3 cortices according to Fischgrund principles), 11/44 (25%) in the IMN group versus 3/9 (33%) in the no-IMN group, the difference between groups was not statistically significant (Fisher’s exact test, *p* = 0.684). In the no-IMN group, all patients (3/3, 100%) who had an insufficient number of cortices developed femoral fractures after EF removal. None of patients with sufficient cortices (0/6) has developed a femoral fracture. The association between the number of cortices and femoral fractures after EF removal in the no-IMN group was statistically significant and very strong (Fisher’s exact test, *p* < 0.05, Cramer’s V = 1.0).

In the IMN group, none of the patients with insufficient cortices have developed femoral fracture (0/11), all three cases have occurred in cases with sufficient corticalization (3/33). There was no statistically significant association between femoral fractures after EF removal and the number of cortices of the regenerate, in the IMN group.

Most patients in both groups presented with regenerate shapes of type 1 or 2 by Li classification (90.7% in the IMN group and 66.7% in the no-IMN group). These types are considered healthy [13]. There was no statistically significant difference in the occurrence of healthy regenerate shape between the IMN and no-IMN groups (Fisher’s exact test, *p* = 0.09). All fractures in the IMN group occurred in patients with type 1 or 2 regenerate shapes. There was no statistically significant difference in femoral fracture rate after EF removal by regenerate shape by Li classification in the IMN group (Fisher’s exact test, *p* = 1.00). In the no-IMN group, 2/3 fractures presented with a type 3 (concave) regenerate shape by Li classification. Statistical analysis showed a significant difference in fracture rate by regenerate shape by Li classification (Fisher’s exact test, *p* = 0.024). Over 90% of patients in the no-IMN group (8/9) and all patients in the IMN group presented with regenerate quality type 9 or 10 by Li classification. Regenerate quality by Li classification was not a factor in fracture risk after EF removal (Fisher’s exact test, *p* > 0.05).

### 3.5. Unplanned Surgeries

In two cases, surgery was needed due to malposition of the intramedullary nail. In both cases malposition of the nail caused discomfort in patient, and required surgical treatment. Both of these patients healed without long-term sequelae or femoral fracture. None of these cases were found in patients with femoral fractures after EF removal. In two cases, the intramedullary nail had to be removed due to infectious complications. Both of these patients healed without long-term sequelae or femoral fracture. One femoral fracture in the IMN group required surgical treatment. In addition, this patient experienced a hip dislocation after nail removal 1 year after EF removal, requiring surgical reduction. These cases involved 5 patients who required 6 unplanned surgeries in the IMN group (5/44, 11.4%). In the no-IMN group, 3 patients underwent a total of 4 unplanned surgeries due to femoral fractures (3/9, 33.3%). Statistical analysis showed a higher OR of unplanned surgical treatment of femoral fractures in patients without intramedullary nailing after EF removal, and the difference was statistically significant (Fisher’s exact test *p* < 0.05, OR 21.5). Odds ratios (OR) for unplanned surgical treatment in the no-IMN group are shown in Table 7.

### 3.6. Infectious Complications

There were 2 cases of infection in patients who underwent intramedullary nailing after EF removal. One in single TEN, and one in Rush nail group. In both cases, we have observed a fistula at the pin tract site. There were no wound infection or fistula at the nail entry. Both patients required surgical treatment (nail removal, soft tissue debridement with fistula resection) and antibiotic treatment. In both cases, infections were successfully treated after nail removal. We routinely checked CRP and WBC levels in all patients. We did not find elevated CRP or WBC levels at the time of EF removal in patients who later developed infectious complications. Moreover, all patients in the IMN group were prescribed antibiotics (trimethoprim/sulfamethoxazole) for 5 days after EF removal with prophylactic intramedullary nailing, including those who have later developed infectious complications.

## 4. Discussion

A key finding of our study is that prophylactic intramedullary nailing was associated with a lower observed rate of surgical treatment for femoral fractures after femoral lengthening with EF (*p* = 0.013). However, prophylactic intramedullary nailing did not significantly reduce fracture rates (*p* = 0.054). We believe our results support using intramedullary nailing as a prophylactic technique after femoral lengthening with EF in CFD. Although the overall fracture rate in the IMN group did not reach statistical significance, the rate of surgically treated fractures was significantly lower. This aligns with Abdelgawad et al., who advocated for prophylactic Rush nailing in CFD patients to mitigate fracture risk [10].

Our study confirms that femoral fracture after lengthening is a frequent complication, occurring in 11.3% of patients overall. However, the incidence was markedly higher in the group without prophylactic intramedullary nailing (no-IMN), reaching 33.3% compared to only 6.8% in the IMN group. When accounting for all unplanned interventions, the IMN group had fewer patients requiring unplanned surgery than the no-IMN group (11.4% vs. 33.3%, Fisher’s exact test *p* = 0.12).

We modified Abdelgawad’s technique by performing intramedullary nailing without prior reaming. We used either Rush nails (preferably ≥ 4.0 mm) or a single TEN, depending on femoral anatomy. No intraoperative fractures occurred, and we encountered no difficulties advancing nails through sclerotic regenerate bone. This suggests that a non-reamed technique, performed under meticulous C-arm guidance, can be as safe and effective as the original method. Two cases in the IMN group (4.5%) required revision due to nail malposition: one cortical protrusion and knee irritation at the suprapatellar recess, and one retrograde migration causing soft tissue irritation. Both patients were treated surgically with Rush nail repositioning. Both complications resulted from technical errors, emphasizing the need for meticulous nail advancement and continuous fluoroscopic monitoring throughout the procedure. There were no long-term sequelae.

Among our cohort, a substantial proportion of patients had insufficient cortices at the time of EF removal (26.4% overall, with no significant differences between groups). In this retrospective series, frame removal in patients with insufficient cortices (<3) was dictated by recalcitrant pin-site infections or profound psychological intolerance of the frame by the child. Poor regenerate morphology at EF removal, according to Fischgrund, occurred at similar rates in both groups: IMN (11/44 patients) and no-IMN (3/9 patients). There was no statistically significant difference in bone regenerate shape between the IMN and no-IMN groups. However, the concave shape of the regenerate, by Li classification, appeared to be associated with a higher risk of femoral fractures in the no-IMN group. Furthermore, the absence of fractures within the regenerate in the IMN group suggests that the nail provides structural protection to the newly formed bone. However, our results do not prove that poor bone regenerate morphology can be compensated for by intramedullary nailing, as we used the same criteria for EF removal in both groups. In the historical no-IMN group, insufficient corticalization according to the Fischgrund principles was associated with femoral fracture risk. However, the limited sample size prevents definitive conclusions regarding its predictive value. Regenerate shape, by Li classification, appeared to affect fracture risk in patients in the no-IMN group, as 2/3 fractures occurred in patients with a concave regenerate shape. However, fractures in the IMN group occurred in patients with sufficient corticalization and a healthy regenerate shape (type 1 or type 2). In those patients, Fischgrund principles and Li classification reveal not useful in predicting femoral fracture risk. Although prophylactic intramedullary nailing appeared to protect against poor bone regenerate morphology, caution is warranted. While all three fractures in the historical no-IMN group required surgery, only one in the IMN group did. This pattern is compatible with a possible protective mechanical role of intramedullary nailing, potentially limiting fracture displacement or secondary deformity. Nevertheless, this interpretation remains speculative because of the small number of events and the retrospective group comparison. Therefore, consider prophylactic nailing an additional stabilization strategy rather than a substitute for adequate regenerate consolidation. We do not advise premature EF removal in CFD.

Consistent with the literature, we observed a trend toward higher fracture rates with greater lengthening magnitudes. Burke et al. identified lengthening over 50 mm as a significant risk factor, while others suggest a threshold of 20–30% of the initial femoral length [5,15]. We noted a temporary knee flexion deficit at EF removal, which resolved by the time of nail removal, as also reported by Bhave et al. [16]. This deficit requires intensive physiotherapy (PT), which may inadvertently contribute to fractures during the early post-EF removal weeks, when the bone is stressed while the range of motion is still restricted. Our findings confirm that most early fractures occur during this period and are rarely associated with significant trauma [5,10]. Regarding weight-bearing in no-IMN patients, we maintain an individualized approach based on radiographic evaluation rather than a unified protocol. As for the IMN group, every patient starts PT with weight-bearing as tolerated protected with crutches, and full weight-bearing is promoted from day 1 post-EF removal. All fractures have occurred in patient at least 6 years old (youngest), up to 11 years old, without any upper limbs impairments—therefore in our opinion capable of using crutches after PT training.

In our study, every patient had their CRP level evaluated 1 day prior to EF removal. In the IMN group, all patients had their CRP level evaluated 1 day prior to nail removal. Elevated CRP levels at EF removal (mean 16.15 mg/L for all patients) likely reflect the localized inflammatory response to percutaneous pins rather than overt infection. In our study, 52% of patients presented with elevated CRP with the EF in situ, dropping to 15% at nail removal one year later. However, we did not find elevated CRP or WBC levels at the time of EF removal in patients who later developed infectious complications. Although CRP and WBC levels were not predictive of later infection, we treated all patients in the IMN group with antibiotics—a 5-day oral regimen of trimethoprim/sulfamethoxazole—and we recommend a delayed approach to nailing if active pin-site infections are present. None of the patients in the no-IMN group received antibiotics. Our data did not identify local inflammation as a primary driver of deep infectious complications. Infectious complications occurred in 4.5% of our IMN cases, matching rates reported by Abdelgawad (7%) and Schiedel (1%) [10,12]. These cases were successfully managed through irrigation, debridement, nail removal, and antibiotics, with no subsequent fractures occurring in these patients.

Ultimately, the 6.8% fracture rate in our IMN group is lower than that reported by Abdelgawad (18% in CFD) and Schiedel (12% in congenital conditions and congenital short stature), though higher than Rovira Marti et al.’s results (2.4%, but exclusively in patients with achondroplasia and hypochondroplasia) [10,11,12]. Abdelgawad et al. reported femoral fractures in 18% of prophylactically rodded cases compared with 34% in a historical cohort without rodding among patients with CFD. Similarly to our results, their data showed that not all femoral fractures in the IMN group required surgical treatment (4 cases, 9% vs. 18% in their study). Similarly, Schiedel et al. reported 12 fractures following 101 femoral lengthening procedures with prophylactic nailing, of which only two required surgical treatment. These studies and ours confirm a high risk of fracture after femoral lengthening in CFD. More recently, Rovira Martí et al. reported fracture rates of 2.4% following prophylactic intramedullary nailing (none required surgical treatment) and 15.9% without nailing in patients with achondroplasia or hypochondroplasia. However, differences in underlying diagnoses, lengthening magnitude, implant characteristics, and postoperative protocols limit direct comparisons. Abdelgawad et al. showed differences in fracture rates by Rush nail diameter (Abdelgawad’s subgroup with 3.18 mm Rush nails showed a 28% fracture rate vs. an 8.3% fracture rate with a 4.76 mm Rush nail). In our study, we used Rush nails of at least 4.0 mm. Schiedel et al., who used one or two TENs, reported a higher fracture rate. In our study, the Rush nail was preferred, while a single TEN was used only in cases of a narrow canal or shaft deformation based on surgeon’s experience. Our retrospective analysis showed a statistically significant difference in nail diameters (Rush nails were bigger), but no statistically significant difference in the percentage of canal fill at the narrowest point. This shows that TENs were chosen in cases with narrower femoral canals. The lowest canal-fill percentages in our study for single TEN and Rush nails were 35% and 34%, respectively. Both exceeded the minimum canal-fill threshold of 15% proposed by Popkov et al. [17]. Fractures in our IMN group that occurred during normal activity within two weeks post-EF removal occurred in patients with 4.0 mm Rush nails. These healed without surgical intervention and without significant angulation or shortening (>5° or >5 mm). Although we found no statistically significant difference in fracture risk between Rush nails and single TENs, the only fracture requiring surgery occurred in a patient with a single TEN. It is important to remember that Rush nails and TENs differ in terms of material (steel vs. titanium), and in our cases, in terms of diameter. Based on our experience and literature we still prefer Rush nail, whenever it is possible to use.

Shadi et al. recently reported additional evidence supporting prophylactic intramedullary nailing after limb lengthening in patients with fibular hemimelia undergoing tibial lengthening [18]. In their retrospective comparative study, no fractures occurred in 24 lengthening procedures followed by prophylactic intramedullary nailing, compared with six fractures in 24 procedures (25%) managed with cast immobilization after external fixator removal (*p* = 0.0229). No infectious complications were observed in either group. Although these findings are consistent with a potential protective role of intramedullary nailing after EF removal, differences in anatomical site, underlying congenital deficiency, postoperative management, and study design limit direct comparison with our femoral lengthening cohort. Nevertheless, the findings from both studies suggest that prophylactic intramedullary nailing may be a useful strategy to reduce post-lengthening fracture risk in selected patients with congenital lower limb deficiencies.

In CFD patients, hip joint dislocation remains a potential risk following Rush nail removal, as presented in our previous study [19]. In the IMN group, hip dislocation occurred in one case after Rush nail removal—one year after EF removal. This required subsequent surgical management. Consequently, we propose routine radiographic hip inspection after nail removal in CFD patients.

It is worth discussing another prophylactic technique for preventing fractures after lengthening: Lengthening Over the Nail (LON). This technique relies on intramedullary nails implanted together with EF. Popkov’s studies showed that LON with a flexible intramedullary nail (FIN) reduces EFI and fracture risk after lengthening in congenital deficiencies [17]. The rate of fractures requiring surgical treatment in Popkov’s study was higher than in our material (4/70, 5.7% vs. 1/44, 2.3% in our study); however, there were 4 cases of unplanned surgeries in our IMN group due to reasons other than fractures (total 5/44, 11.4% of unplanned surgical treatment). To reliably compare these two approaches in congenital deficiencies (LON and prophylactic nailing after EF removal), a randomized controlled trial (RCT) is required. There are advantages of LON—especially in patients with compromised bone formation. LON, with the use of FIN, allows hydroapatite-coated nails, which improve outcomes in cases with abnormal bone (metabolic bone disorders or skeletal dysplasias) [17,20,21]. A case series by Mingazov et al. showed that femoral lengthening by LON is possible using a telescopic intramedullary nail [22].

Additional evidence regarding the role of intramedullary nailing in distraction osteogenesis comes from studies involving bone transport. Wan et al. retrospectively compared femoral bone transport using a monolateral external fixator alone or combined with an intramedullary nail [23]. They reported fewer overall complications and a shorter external fixation duration in the combined fixation group, although two patients required amputation due to persistent deep infection. These observations support the potential role of intramedullary nailing in maintaining mechanical stability during regenerate consolidation. However, bone transport for acquired segmental defects differs substantially from femoral lengthening in children with congenital femoral deficiency.

### Study Limitations

There are several limitations to this study. First, the small cohort size is constrained by the rarity of the procedure and the congenital deformity. Nevertheless, our cohort is homogeneous and includes all consecutively treated patients with a single underlying condition (CFD) at a single center over 10 years of practice. Cohort size is in line with other authors, mentioned in the discussion, who reports similar sample sizes (Abdelgawad et al. with 43 CFD patients, Prince et al. with 38 CFD patients, Szymczuk et al. with 62 patients with congenital deformities, or Popkov et al. with 70 patients with congenital deformities, but 19 with monofocal femoral lengthening), underscoring that small sample size is a troubling limitation for congenital deformities research worldwide [3,10,17,24]. Second, there is heterogeneity in EF fixator types and group sizes. These limitations reflect clinical practice driven by the literature, which suggests greater comfort with external lateral fixators and better outcomes with prophylactic measures to prevent fractures after femoral lengthening (such as prophylactic nailing or lengthening over the nail) [3,9,10,11,12,17]. Moreover, our outcomes improved during the observation period. As we observed fewer fractures requiring surgical treatment after femoral lengthening with EF in CFD with prophylactic intramedullary nailing, we shifted toward performing it routinely. Currently, at our institution, prophylactic intramedullary nailing following femoral lengthening with EF in CFD is the preferred treatment. This treatment preference changed in 2017, introducing selection bias in our study. Therefore, the CFD group treated without prophylactic nailing is small and reflects bias associated with the study’s retrospective design. However, the nine patients in the no-IMN group comprise the complete, unselected historical cohort of consecutive CFD patients treated in the department before the shift in the preferred treatment. After prophylactic nailing became the preferred standard of care, we no longer performed no-IMN cases, which accounts for the difference in group sizes. Group heterogeneity in EF fixator type is another limitation of our study. The shift toward monolateral frames coincided with the transition to routine prophylactic nailing after EF removal, making it difficult to distinguish the potential effects of intramedullary nailing from other treatment-related factors. The groups also differed in follow-up duration. Although exploratory subgroup analyses did not detect statistically significant associations between EF type and fracture occurrence, the small subgroup sizes do not establish equivalence between EF types or rule out residual confounding. To match control and study groups and address the limitation of small sample size, a prospective randomized controlled trial (RCT), preferably multicenter, is needed. Still, the outcomes in CFD patients in the IMN group suggest a potential clinical benefit of prophylactic nailing. Lastly, a single investigator (JT) assessed all radiological parameters; therefore, the study did not formally evaluate inter- and intra-observer reliability.

Finally, while internal motorized or magnetic nails are increasingly common and bypass the need for secondary protective procedures, EF lengthening remains essential for young patients with narrow intramedullary canals [24,25,26]. In these cases, particularly those with congenital etiologies, prophylactic intramedullary nailing after EF removal is a potentially useful strategy for favorable outcomes.

## 5. Conclusions

In this retrospective comparative study with historical controls, intramedullary nailing after femoral lengthening with EF in CFD was associated with a lower observed rate of fractures requiring surgical treatment after EF removal. Implant-related infection occurred in 4.5% of patients and required surgical treatment, highlighting the need to balance potential benefits against procedural risks.

## Figures and Tables

**Table 1:** Patients’ characteristics.

	IMN Group	No-IMN Group	Total	*p*
	*N* = 44	*N* = 9	*N* = 53	
Gender				
male	25	2	27	*p* > 0.05
female	19	7	26	*p* > 0.05
EF type				
monolateral	40	3	43	*p* < 0.05
circular	4	6	10	*p* < 0.05
Age at lengthening (in years)	8.8	10.8	9.2	*p* > 0.05
Follow-up after EF removal (in months)	37.2	70.8	42.9	*p* < 0.05

IMN—Intramedullary nailing, EF—External Fixator.

**Table 2:** Characteristics of fracture cases in IMN group.

Case	Etiology of Discrepancy	Weight-Bearing After EF Removal	Nail Type	Timing of Fracture	Mechanism of Fracture	Fracture Type [7]	Treatment
1	CFD	Full with crutches	Rush	1 day post removal	Low-energy/daily activity	Screw track (type III)	Non-surgical
2	CFD	Full with crutches	Rush	14 days post removal	Low-energy/daily activity	Screw track (type III)	Non-surgical
3	CFD	Full with crutches	TEN	4 months post removal	Traumatic	Screw track (type III)	Surgical

EF—External Fixator, CFD—Congenital Femoral Deficiency, IMN—Intramedullary nailing.

**Table 3:** Characteristics of femur fracture cases in no IMN group.

Case	Etiology of Lower Limb Discrepancy	Weight-Bearing After EF Removal	Timing of Fracture After EF Removal	Mechanism of Fracture	Fracture Type [7]	Treatment
1	CFD	None with crutches	7 day	Low-energy/daily activity	Within the regenerate zone (type I)	Surgical
2	CFD	Partial with crutches	1 day	Low-energy/daily activity	Within the regenerate zone (type I)	Surgical
3	CFD	Partial with crutches	14 day	Low-energy/daily activity	Junctional (type II)	Surgical

CFD—Congenital Femoral Deficiency, EF—External Fixator, IMN—Intramedullary nailing.

**Table 4:** Femoral lengthening parameters in the IMN vs. the no-IMN group.

Femoral Lengthening Parameter	Intramedullary Nailing (*n* = 44)	No Intramedullary Nailing (*n* = 9)	*p*
Achieved lengthening in cm	5.66 (SD = 1.18)	5.47 (SD = 1.69)	>0.05
Achieved lengthening in % of femur length	22.00 (SD = 9.17)	19.71 (SD = 10.34)	>0.05
External Fixator Time (days)	214 (SD = 42)	211 (SD = 56)	>0.05
External Fixator Index (days/cm)	40 (SD = 13)	42 (SD = 17)	>0.05

IMN—Intramedullary nailing.

**Table 5:** Femoral lengthening parameters in fractured vs. non-fractured patients.

Femoral Lengthening Parameter	Fracture (*n* = 6)	No Fracture (*n* = 47)	*p*
Achieved lengthening in cm	5.78 (SD = 1.39)	5.61 (SD = 1.26)	>0.05
Achieved lengthening in % of femur length	25.27 (SD = 10.67)	21.12 (SD = 9.15)	>0.05
External Fixator Time (days)	212 (SD = 74)	214 (SD = 40)	>0.05
External Fixator Index (days/cm)	38 (SD = 13)	40 (SD = 14)	>0.05

**Table 6:** Knee range of flexion at EF removal (fractured vs. non-fractured) and at nail removal in the IMN group.

Knee Range of Flexion	At EF Removal (All)	At EF Removal (Fractured)	At EF Removal (Non-Fractured)	At Nail Removal	*p*
Mean in degrees (SD)	36.0 (35.0)	40 (40)	35.7 (34.7) ^+^	109 (39.9) *	* <0.001

EF—External Fixator. * significant difference between at EF removal vs. at nail removal. ^+^ not significant difference between at EF removal (fractured) vs. at EF removal (non-fractured).

**Table 7:** Unplanned surgical treatment in the IMN vs. the no-IMN group.

Unplanned Surgeries				
	IMN Group (*n* = 44)	No IMN Group (*n* = 9)	OR	*p*
Removal due to infection	2 (4.5%)	0		
Removal due to malposition	2 (4.5%)	0		
Hip dislocation after nail removal	1 (2.3%)	0		
ORIF + CRIF	1 (2.3%)	3 (33.3%)	21.5	0.013
Patients with unplanned surgery	5 (11.4%)	3 (33.3%)	3.90	0.12

IMN—Intramedullary nailing, ORIF—Open Reduction and Internal Fixation, CRIF—Closed Reduction and Internal Fixation.

## Data Availability

The data that support the findings of this study are included within the article. The raw data are not publicly available but can be accessed by researchers upon reasonable request from the corresponding author, Jędrzej Tschurl (jtschurl@orsk.pl).

## Abbreviations

The following abbreviations are used in this manuscript:

EF	External Fixator
CFD	Congenital Femoral Deficiency
IMN	Intramedullary Nailing
TEN	Titanium Elastic Nail
OR	Odds Ratio
CRIF	Closed Reduction and Internal Fixation
ORIF	Open Reduction and Internal Fixation
